# Surgical Valvotomy Versus Balloon Valvuloplasty for Congenital Aortic Valve Stenosis: A Systematic Review and Meta‐Analysis

**DOI:** 10.1161/JAHA.116.003931

**Published:** 2016-08-08

**Authors:** Garick D. Hill, Salil Ginde, Rodrigo Rios, Peter C. Frommelt, Kevin D. Hill

**Affiliations:** ^1^ Division of CardiologyDepartment of PediatricsMedical College of WisconsinMilwaukeeWI; ^2^ Division of CardiologyDepartment of PediatricsDuke UniversityDurhamNC

**Keywords:** aortic surgery, aortic valve stenosis, balloon aortic valvuloplasty, congenital heart defects, meta‐analysis, Cardiovascular Surgery, Catheter-Based Coronary and Valvular Interventions

## Abstract

**Background:**

Optimal initial treatment for congenital aortic valve stenosis in children remains unclear between balloon aortic valvuloplasty (BAV) and surgical aortic valvotomy (SAV).

**Methods and Results:**

We performed a contemporary systematic review and meta‐analysis to compare survival in children with congenital aortic valve stenosis. Secondary outcomes included frequency of at least moderate regurgitation at hospital discharge as well as rates of aortic valve replacement and reintervention. Single‐ and dual‐arm studies were identified by a search of PubMed (Medline), Embase, and the Cochrane database. Overall 2368 patients from 20 studies were included in the analysis, including 1835 (77%) in the BAV group and 533 (23%) in the SAV group. There was no difference between SAV and BAV in hospital mortality (OR=0.98, 95% CI 0.5–2.0, *P*=0.27, I^2^=22%) or frequency of at least moderate aortic regurgitation at discharge (OR=0.58, 95% CI 0.3–1.3, *P*=0.09, I^2^=54%). Kaplan–Meier analysis showed no difference in long‐term survival or freedom from aortic valve replacement but significantly more reintervention in the BAV group (10‐year freedom from reintervention of 46% [95% CI 40–52] for BAV versus 73% [95% CI 68–77] for SAV,* P*<0.001). Results were unchanged in a sensitivity analysis restricted to infants (<1 year of age).

**Conclusions:**

Although higher rates of reintervention suggest improved outcomes with SAV, indications for reintervention may vary depending on initial intervention. When considering the benefits of a less‐invasive approach, and clinical equipoise with respect to more clinically relevant outcomes, these findings support the need for a randomized controlled trial.

## Introduction

Optimal initial treatment for congenital aortic valve stenosis in children remains controversial. Ideally, initial intervention would achieve adequate relief of obstruction without causing significant regurgitation. Balloon aortic valvuloplasty (BAV) and surgical aortic valvotomy (SAV) represent competing strategies, and the choice of primary intervention is typically based on institutional preference.

A 2001 landmark analysis of the Congenital Heart Surgeons’ Society database demonstrated equivalent outcomes in regard to survival and need for reintervention for BAV compared to SAV in 110 neonates across 18 institutions.^1^ Given similar outcomes, many have considered catheter‐based balloon valvuloplasty to be a more attractive option as it is less invasive with shorter postprocedural recovery. However, recent single‐center analyses have shown better outcomes with surgical valvotomy. These improved outcomes have been attributed to the use of contemporary surgical approaches using more precise techniques.^2^, ^3^ To date no randomized trial has compared BAV and SAV, and it is unlikely such a trial will provide answers in the near future, as long‐term follow‐up is needed for comparison.

In light of the current controversy surrounding the optimal initial strategy for intervention, we performed a contemporary systematic review and, using pooled data from single‐ and dual‐arm studies, conducted a meta‐analysis. Our primary objective was to determine whether BAV or SAV as primary intervention for congenital aortic valve stenosis in pediatric patients had superior outcomes in regard to rates of reintervention, aortic valve replacement, and survival.

## Methods

Studies were identified in PubMed (Medline), Embase, and the Cochrane databases by an experienced librarian using search terms of: {(((“Heart Defects, Congenital”[Mesh]) AND (“Aortic Valve Stenosis”[Mesh])) OR ((congenital aortic valve stenosis) OR congenital aortic stenosis)) OR ((congenital) AND “Aortic Valve Stenosis”[Mesh])) OR ((“Heart Defects, Congenital”[Mesh]) AND “Heart Valves”[Mesh])} AND {((aortic dilation) OR catheter balloon angioplasty) OR ((“Cardiac Catheters”[Mesh]) OR ((balloon valvuloplasty) OR valvuloplasty) AND “Balloon Valvuloplasty”[Mesh])} OR {(bicuspid valve repair) OR ((valvotomy) OR surgical repair)}. The final search was performed on March 17, 2016. The reference lists of all reviewed full‐text articles were also evaluated for additional articles.

**Figure 1:**
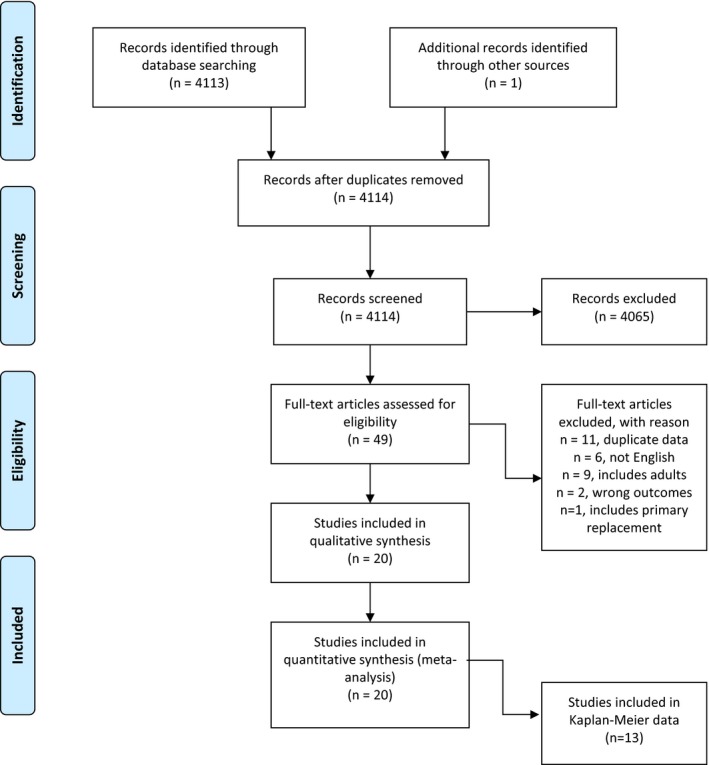
Flow diagram of study selection.

Because our primary objective was to evaluate contemporary outcomes, we only included studies published after 2000. This search strategy identified 4113 studies with 1 additional article identified in the reference list search. Abstracts from potentially relevant articles were evaluated by a pediatric cardiologist (G.D.H.) to determine eligibility for inclusion in the analysis. All studies reporting outcomes of either BAV and/or SAV as primary intervention for aortic stenosis in children/adolescents ≤18 years of age at the time of intervention were included. Studies including patients >18 years of age or valve replacement as initial treatment were included only if those patients could be eliminated from the analysis. Studies reporting fewer than 10 patients, studies not published in English, and those that were only abstracts from scientific meetings without a published manuscript were excluded. When multiple studies were reported from the same institution with potentially overlapping patients, the most recent study was used. After application of the inclusion/exclusion criteria, 20 studies were eligible for the meta‐analysis (Figure 1).^1^, ^2^, ^3^, ^4^, ^5^, ^6^, ^7^, ^8^, ^9^, ^10^, ^11^, ^12^, ^13^, ^14^, ^15^, ^16^, ^17^, ^18^, ^19^, ^20^ Two database studies were included that may have overlapping patients with other single‐center reports.^1^, ^8^

Study quality was assessed independently by 2 reviewers (R.R., S.G.) using the Hayden bias‐rating tool. This scale rates 6 domains (study participation, study attrition, prognostic factor measurement, outcome measurement, study confounding, and statistical analysis and reporting) for their risk of bias.^21^ The rating was converted to a numeric scale (3=high risk of bias, 2=moderate risk of bias, 1=low risk of bias) for each of the domains with a minimum possible total score of 6 (lowest risk of bias) and maximum possible total score of 18 (highest risk of bias). Interrater reliability was assessed using the Cohen κ coefficient. Data extracted by a single author (G.D.H) included subject characteristics including age at intervention, number of neonates (≤30 days of age at intervention) and infants (<1 year of age), valve morphology (unicuspid, bicuspid, or tricuspid), peak preintervention echocardiographic Doppler gradient, and outcomes of intervention (including number with moderate or greater regurgitation at discharge, hospital or 30‐day mortality, and peak postintervention gradient). In addition to these outcomes, Kaplan‐Meier data were extracted from a subset of 13 studies with available data on long‐term survival, time to reintervention, and/or time to aortic valve replacement. Reintervention included repeat BAV, SAV, or valve replacement, but most studies did not differentiate SAV from BAV reinterventions. For this analysis we used the method described by Guyot et al to recreate individual patient data by distributing censoring evenly over intervals where numbers at risk were provided.^22^ We used Plot Digitizer 2.6.6 to extract coordinates from curves. For some studies, data could not be extracted accurately by this method because numbers at risk were not provided or because of different grouping of patients. In these cases authors were contacted, and individual patient data from 2 of these studies were provided by the study authors.^15^, ^18^ Data were pooled using the Mantel‐Haenszel random‐effects model, and heterogeneity was assessed using the I^2^ method. Pooled categorical comparisons were made using a Chi‐squared test. For all analyses our primary outcome of interest was survival, which was assessed in the short‐term (hospital/30‐day survival) and at longer‐term follow‐up. Secondary outcomes included at least moderate regurgitation at hospital discharge, long‐term freedom from aortic valve replacement, and freedom from reintervention. For comparisons of short‐term outcomes, all noncomparative studies were pooled into a single study to generate comparisons of hospital/30‐day survival and at least moderate regurgitation at discharge. For longer‐term outcomes, extracted and author‐supplied individual patient data were used to generate Kaplan‐Meier curves for survival, freedom from aortic valve replacement, and freedom from reintervention. As a sensitivity analysis, Kaplan‐Meier curves for survival, freedom from aortic valve replacement, and freedom from reintervention were also created using studies limited to infants (<1 year) at the time of BAV or SAV and studies in which individual patient data could limit inclusion to this age group. Meta‐regression including overall Hayden risk of bias score was performed for all hospital outcomes. Kaplan‐Meier curves for survival, freedom from aortic valve replacement, and freedom from reintervention were also stratified by the Hayden risk of bias score with separate curves for those at lowest risk of bias (score of 6) and those at higher risk created to ensure results were similar. Publication bias was assessed visually with a funnel plot. Data analysis was performed using Stata 13 (Stata Corp, College Station, TX) with *P*<0.05 considered significant.

## Results

Overall, 2368 patients were included in the analysis including 1835 (77%) in the BAV group and 533 (23%) in the SAV group. Studies included in the analysis and those with data appropriate for Kaplan‐Meier data extraction along with Hayden bias scores can be seen in Table 1. Hayden scores ranged from 6 to 9. Full scoring by Hayden bias domain can be seen in Table S1. There was 97% agreement and a κ coefficient of 0.73 for interrater reliability.

**Table 1:** Included Studies

Article	Year	Location	Intervention (n)	Hayden Score	KM Data
Alexiou^4^	2001	Southamptom, UK	SAV (97)	7	Extracted
Bhabra^5^	2003	Birmingham, UK	SAV (54)	6	Extracted
Brown^2^	2012	Indiana	BAV (69) SAV (89)	7	Extracted
Crespo^6^	2009	Montreal, Canada	BAV (143)	7	Extracted
Elshershari^7^	2002	Ankara, Turkey	SAV (28)	9	No KM data
Ewert^8^	2011	Multi—Germany, Austria, Switzerland	BAV (1004)	6	Number at risk not provided
Hamidi‐Manesh^9^	2013	London, UK	BAV (29)	7	No KM data
Han^10^	2007	Toronto, Canada	BAV (53)	6	Extracted
Hochstrasser^11^	2015	Lausanne, Switzerland	BAV (22) SAV (42)	7	Extracted
Jindal^12^	2000	New Delhi, India	BAV (10)	6	No KM data
Kim^13^	2005	Atlanta	BAV (20)	6	No KM data
Latiff^14^	2003	Sydney, Australia	BAV (42)	6	Extracted
Loomba^15^	2015	Multi—Milwaukee, Rochester	BAV (50) SAV (52)	6	Author provided
McCrindle^1^	2001	Multi—Congenital Heart Surgeons Society	BAV (82) SAV (28)	6	Number at risk not provided
McElhinney^16^	2005	Boston, MA	BAV (113)	7	Extracted
Miyamoto^17^	2006	Sankt Augustin, Germany	SAV (34)	6	Extracted
Prijic^18^	2014	Belgrade, Serbia	BAV (39) SAV (23)	6	Author provided
Robinson^19^	2000	Multi—Pittsburgh, Hershey, Gainesville, Warsaw Poland	BAV (92)	6	Number at risk not provided
Rossi^20^	2011	Porto Alegre, Brasil	BAV (30)	6	In article
Siddiqui^3^	2013	Melbourne, Australia	BAV (37) SAV (86)	6	Extracted

Studies included in the systematic review by first author name with year of publication and location. Hayden risk of bias scale is 6 (low risk of bias) to 18 (high risk of bias). BAV indicates balloon aortic valvuloplasty; KM, Kaplan‐Meier; SAV, surgical aortic valvotomy.

Overall mean age at intervention was 2.9 (95% CI −0.7 to 6.4) months with a mean peak preintervention systolic Doppler gradient of 77 (95% CI 66–88) mm Hg. There were no significant differences between the BAV and SAV groups with respect to these 2 variables (Table 2). There were differences between groups in the amount of reported data: specifically, age group was more often reported in the BAV group, and valve morphology was more commonly reported in the SAV group. In those studies reporting data for age group, there was a higher percentage of children age ≥1 year in the BAV group (33% for BAV versus 24% for SAV, *P*=0.03). In those studies reporting data for valve morphology, unicuspid aortic valve was more common in the BAV group (*P*=0.02).

**Table 2:** Baseline Characteristics

	SAV (n=533)	BAV (n=1835)	*P* Value
Mean age (95% CI), months	8.4 (−5.2 to 22.0)	2.1 (−1.6 to 5.9)	0.73
Age group			
Reported	247 (46%)	1566 (85%)	<0.0001
Neonates (≤30 days)	121 (49%)	712 (45%)	0.03
Infants (<1 year)	66 (27%)	345 (22%)	
Children (≥1 year)	60 (24%)	509 (33%)	
Valve morphology			
Reported	323 (61%)	402 (22%)	<0.0001
Unicuspid	14 (4%)	38 (9%)	0.02
Bicuspid	238 (74%)	292 (73%)	
Tricuspid	71 (22%)	72 (18%)	
Prepeak echo gradient (95% CI), mm Hg	74 (60–89)	81 (63–99)	0.17

Comparison of preoperative factors by group. BAV indicates balloon aortic valvuloplasty; SAV, surgical aortic valvotomy.

### Hospital/Short‐Term Outcomes

**Figure 2:**
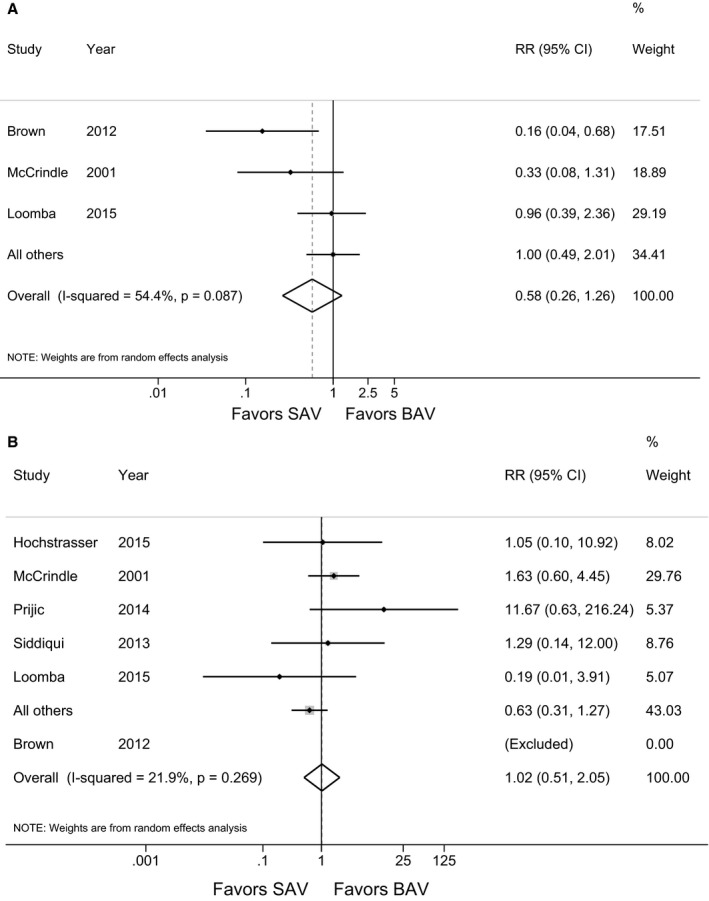
Forest plot comparing incidence of moderate or greater aortic valve regurgitation prior to discharge or at early postoperative follow‐up (A) and hospital or 30‐day mortality (B) by intervention. The Brown study was excluded from mortality plot because there were no deaths in either group. Noncomparative studies of SAV and BAV were combined as “All others.” BAV indicates balloon aortic valvuloplasty; SAV surgical aortic valvotomy.

There was no difference in postintervention peak systolic Doppler gradient (28 mm Hg [95% CI 19–37] for SAV versus 37 mm Hg [95% CI 26–49] for BAV, *P*=0.06) nor frequency of at least moderate aortic regurgitation at discharge with SAV versus BAV (OR=0.58, 95% CI 0.3–1.3, *P*=0.09). However, there was significant heterogeneity between results (I^2^=54%) reflecting variability between studies (Figure 2A). In studies reporting hospital or 30‐day mortality, there were 21 deaths out of 533 (4%) SAV patients and 105 deaths out of 1815 (6%) BAV patients with no difference in hospital or 30‐day mortality between SAV and BAV (OR=0.98, 95% CI 0.5–2.0, *P*=0.27, I^2^=22%) (Figure 2B). Overall Hayden risk of bias score did not reach significance for either mortality (*P*=0.14) or risk of at least moderate regurgitation (*P*=0.08).

### Long‐Term Outcomes

**Figure 3:**
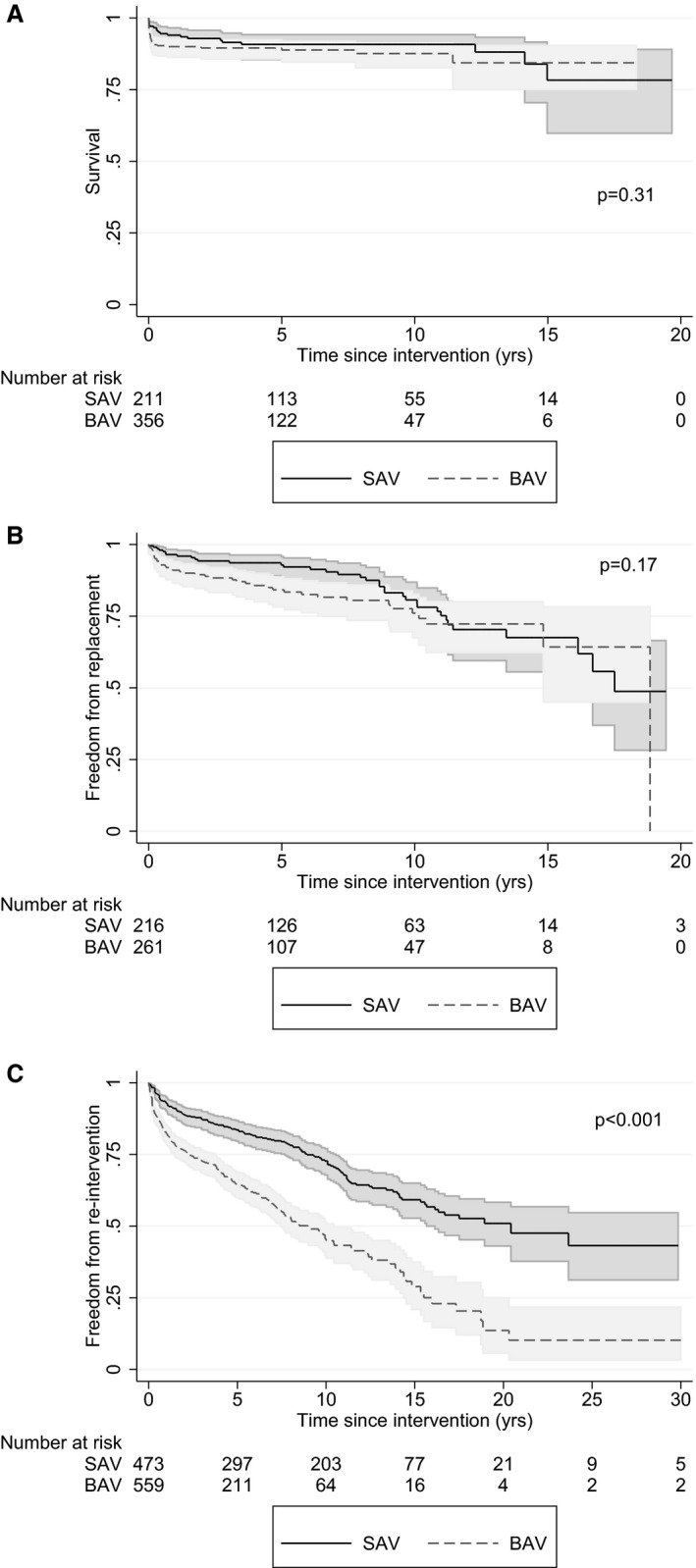
Kaplan‐Meier curves for survival (A), freedom from aortic valve replacement (B), and freedom from reintervention (C) by intervention in all patients <18 years of age. BAV indicates balloon aortic valvuloplasty; SAV surgical aortic valvotomy.

Kaplan‐Meier curves showed no significant difference in survival between groups (*P*=0.31) (Figure 3A). Survival at 10 years was 87% (95% CI 81–90) in the BAV group and 90% (95% CI 85–94) in the SAV group. In terms of secondary outcomes measures, freedom from valve replacement was also not significantly different between groups (*P*=0.17) (Figure 3B). Ten‐year freedom from valve replacement was 76% (95% CI 67–83) for BAV and 81% (95% CI 72–87) for SAV. However, there was significantly more reintervention in patients undergoing initial BAV compared to SAV (*P*<0.001) (Figure 3C). Freedom from reintervention at 10 years was 46% (95% CI 40–52) in the BAV group and 73% (95% CI 68–77) in the SAV group.

**Figure 4:**
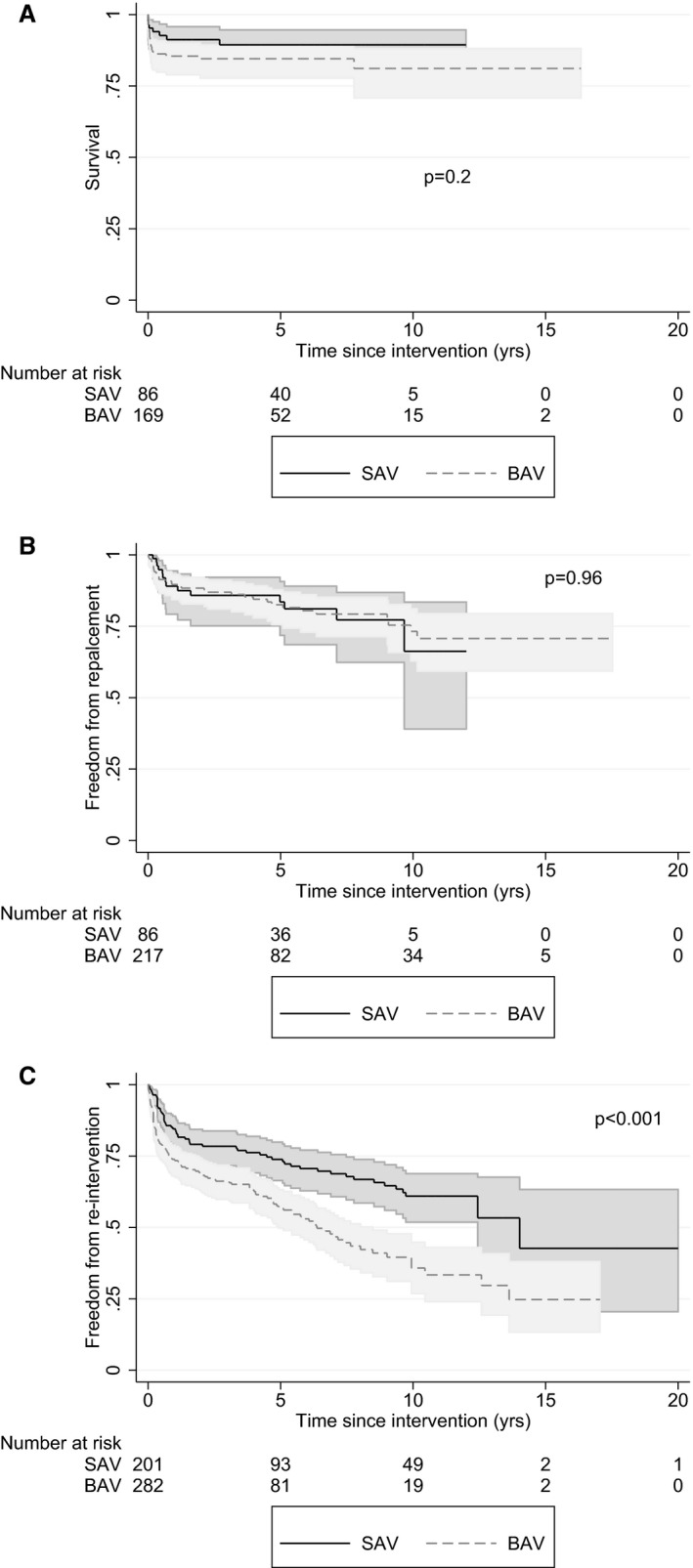
Kaplan‐Meier curves for survival (A), freedom from aortic valve replacement (B), and freedom from reintervention (C) by intervention in infants <1 year of age at initial intervention. BAV indicates balloon aortic valvuloplasty; SAV, surgical aortic valvotomy.

In sensitivity analysis restricted to the subset of infants (<1 year of age) at the time of BAV (n=282) or SAV (n=201), results were unchanged, with no difference between groups in survival (*P*=0.23) or freedom from valve replacement (*P*=0.7) (Figure 4A and 4B), but there was more reintervention in the BAV group (*P*<0.001) (Figure 4C). There was no difference in results when they were stratified by overall Hayden risk of bias score (Figure S1).

## Discussion

In this meta‐analysis comparing balloon valvuloplasty and surgical valvotomy for children presenting with congenital aortic valve stenosis, we demonstrate no differences in long‐term survival or rates of aortic valve replacement but significantly higher rates of reintervention following balloon valvuloplasty. The findings were unchanged when the analysis was limited to infants (<1 year of age) at initial intervention.

The choice of BAV or SAV has to date been based on little comparative data. We identified only 6 comparative studies published since 2000.^1^, ^2^, ^3^, ^11^, ^15^, ^18^ Five of these represent single‐ or dual‐center reports with patients treated with either SAV or BAV.^2^, ^3^, ^11^, ^15^, ^18^ Conclusions ranged from clear benefit with SAV^2^, ^3^ to similar outcomes with either approach.^18^ The sixth study, a landmark study by McCrindle et al, was a database analysis including 110 neonates from 18 centers participating in the Congenital Heart Surgeons’ Society (CHSS) database between 1994 and 2000. In multivariable analysis they found no difference in freedom from reintervention or survival between BAV and SAV.^1^ However, in a more recent analysis, Siddiqui et al demonstrated significantly better freedom from reintervention and aortic valve replacement after SAV. In that study the authors argued that improvements in surgical technique over time, including thinning of leaflets and resection of nodular dysplasia, could account for the differences noted.^3^ Our findings are similar to those reported by Siddiqui et al with increased reintervention after BAV, although it should be noted that our pooled analysis demonstrates substantially lower rates of reintervention after BAV (54% in our pooled analysis versus 73% in the Siddiqui et al analysis at 10 years).

For several reasons it could be argued that the reintervention rate represents a suboptimal outcome measure for congenital aortic valve stenosis. First, decisions to reintervene are typically arbitrary with no uniform criteria, particularly when there is mixed valvar disease with both stenosis and insufficiency. Second, thresholds for reintervention may vary depending on the institutional preference for BAV versus SAV. Finally, center approach may vary as BAV potentially allows for a staged approach, either as a means to delay until SAV or with more frequent but more “gentle” dilation of the valve, thereby leaving more stenosis but avoiding regurgitation. The majority of patients with residual/recurrent stenosis after BAV are treated with repeat BAV, whereas those with insufficiency or mixed stenosis and insufficiency are treated surgically, typically with valve replacement.^23^, ^24^ Notably, our data do not entirely fit this latter hypothesis as, although not quite statistically significant, there was more aortic regurgitation after BAV (*P*=0.09), and patients demonstrated greater residual echocardiographic peak Doppler gradients (*P*=0.06). In any case, the benefit of this meta‐analysis is that it provides enough patients to power comparison of more meaningful but less common outcomes such as mortality and valve replacement. When focusing on these more concrete endpoints, our findings demonstrated no difference between the competing strategies and were reassuring overall, with pooled survival rates of 85% to 90% at 10 years. Nonetheless, it is clear that this represents a lifelong disease. Both reintervention and valve replacement were common, occurring in 40% and 20% to 25%, respectively, by 10 years after initial intervention. These data highlight that for many patients both BAV and SAV can be considered as palliative procedures, delaying rather than preventing valve replacement.

A novel aspect of this study is that we used a method to extract individual patient data from published Kaplan‐Meier curves. This method was published in 2012 by Guyot et al and involves distributing censored events evenly over the time period between provided numbers at risk. This method is accurate with a mean absolute error of −0.272% for survival estimates if all information (total number of events and numbers at risk) is provided.^22^ We chose to exclude studies without all information as the accuracy of extracted data deteriorates. In this way we were able to recreate individual patient data and combine data from comparative studies with data from single‐arm studies of SAV^4^, ^5^, ^7^, ^17^ and BAV.^6^, ^8^, ^9^, ^10^, ^12^, ^13^, ^14^, ^16^, ^19^, ^20^

Despite our novel approach, this study is nonetheless limited by the fact that patients were not randomized or even in comparative studies. Some studies had unreported data regarding age at initial intervention, valve morphology, and other baseline characteristics. Moreover, uniformity of outcome definitions between studies is often problematic in meta‐analyses. In this study reintervention was the only variable outcome measure. It was defined by the majority as any repeat aortic valve procedure (including BAV, SAV, or aortic valve replacement)^2^, ^3^, ^4^, ^5^, ^10^, ^11^, ^15^, ^16^, ^17^, ^18^, ^20^; however, studies typically did not specify whether BAV or SAV was used for reintervention. Moreover, 2 studies also included non–aortic valve reinterventions. We elected to include these studies as these reinterventions (which could not be individually excluded) represented only 8 cases from the BAV group out of the 67 total reinterventions and 185 patients included from these 2 studies.^6^, ^14^ Because of these limitations and for the reasons outlined in our earlier discussion, we feel that reintervention rate is a less meaningful study endpoint than the “harder” endpoints of survival or time to aortic valve replacement. Another limitation is the possibility that patients from different studies may have different baseline characteristics. To reduce heterogeneity, we used a sensitivity analysis restricting the cohort to the subset of infants undergoing initial intervention with BAV or SAV. Prior studies have also separately evaluated neonates with critical aortic stenosis^1^; however, we did not have adequate power for this analysis. Additionally, as with any systematic review and meta‐analysis, this study is subject to publication bias. However, we compared noncomparative single‐intervention studies that are subject to the same publication bias, in essence comparing the best results of BAV to the best results of SAV. In visual evaluation of a funnel plot there was no evidence of publication bias. Systematic reviews and meta‐analyses are also limited by the quality of study included. In this case there are many single‐center studies, which may introduce institutional biases. This is particularly true with regard to criteria for reintervention, which was not predetermined in any study. Finally, we used a method to extract data rather than relying on actual individual patient data. Although using individual patient data for meta‐analyses is ideal, it is rarely possible. Data are often no longer available, or authors are unwilling to share their work. The method used in our study has been demonstrated to be remarkably accurate when the total number of events and the number at risk are both presented, as they were in all articles used for these data.^22^

## Conclusions

For children with congenital aortic valve stenosis, initial treatment with balloon valvuloplasty or surgical valvotomy results in similar short‐term gradient reduction, incidence of moderate or greater aortic regurgitation, and survival. In longer‐term follow‐up, there is no difference in freedom from aortic valve replacement or survival in either children or infants. There is a significantly higher rate of reintervention following initial balloon valvuloplasty overall and in infants alone. These data support the use of either approach for initial treatment of children with aortic valve stenosis, based on institutional preference. More importantly these data support equipoise for a randomized trial and also demonstrate the particular importance of capturing as trial endpoints both indication for reintervention and type of reintervention.

## Sources of Funding

This publication was supported by the National Center for Advancing Translational Sciences, National Institutes of Health, through grant number 8UL1TR000055. Its contents are solely the responsibility of the authors and do not necessarily represent the official views of the NIH.

## Disclosures

None.

## Supporting information


**Table S1.** Full Hayden Risk of Bias Scoring
**Figure S1.** Outcomes by risk of bias. Kaplan‐Meier curve for survival (A and B), freedom from aortic valve replacement (C and D), and freedom from reintervention by Hayden risk of bias score (E and F). Scores were dichotomized to low risk (Hayden bias score of 6) or high risk (Hayden bias score >6).

## References

[1] McCrindle BW , Blackstone EH , Williams WG , Sittiwangkul R , Spray TL , Azakie A , Jonas RA . Are outcomes of surgical versus transcatheter balloon valvotomy equivalent in neonatal critical aortic stenosis? Circulation. 2001;104:I152–I158. 11568048 10.1161/hc37t1.094837

[2] Brown JW , Rodefeld MD , Ruzmetov M , Eltayeb O , Yurdakok O , Turrentine MW . Surgical valvuloplasty versus balloon aortic dilation for congenital aortic stenosis: are evidence‐based outcomes relevant? Ann Thorac Surg. 2012;94:146–153; discussion 153‐155. 22537535 10.1016/j.athoracsur.2012.02.054

[3] Siddiqui J , Brizard CP , Galati JC , Iyengar AJ , Hutchinson D , Konstantinov IE , Wheaton GR , Ramsay JM , d'Udekem Y . Surgical valvotomy and repair for neonatal and infant congenital aortic stenosis achieves better results than interventional catheterization. J Am Coll Cardiol. 2013;62:2134–2140. 23954309 10.1016/j.jacc.2013.07.052

[4] Alexiou C , Chen Q , Langley SM , Salmon AP , Keeton BR , Haw MP , Monro JL . Is there still a place for open surgical valvotomy in the management of aortic stenosis in children? The view from Southampton. Eur J Cardiothorac Surg. 2001;20:239–246. 11463538 10.1016/s1010-7940(01)00813-2

[5] Bhabra MS , Dhillon R , Bhudia S , Sethia B , Miller P , Stumper O , Wright JG , De Giovanni JV , Barron DJ , Brawn WJ . Surgical aortic valvotomy in infancy: impact of leaflet morphology on long‐term outcomes. Ann Thorac Surg. 2003;76:1412–1416. 14602259 10.1016/s0003-4975(03)01028-2

[6] Crespo D , Miro J , Vobecky SJ , Poirier N , Lapierre C , Zhao NN , Dahdah N . Experience in a single centre with percutaneous aortic valvoplasty in children, including those with associated cardiovascular lesions. Cardiol Young. 2009;19:372–382. 19519967 10.1017/S1047951109990308

[7] Elshershari H , Alehan D , Demircin M , Pasaoglu I , Bilgic A . Surgical outcome of congenital valvar aortic stenosis. Turk J Pediatr. 2002;44:304–311. 12458805

[8] Ewert P , Bertram H , Breuer J , Dahnert I , Dittrich S , Eicken A , Emmel M , Fischer G , Gitter R , Gorenflo M , Haas N , Kitzmuller E , Koch A , Kretschmar O , Lindinger A , Michel‐Behnke I , Nuernberg JH , Peuster M , Walter K , Zartner P , Uhlemann F . Balloon valvuloplasty in the treatment of congenital aortic valve stenosis—a retrospective multicenter survey of more than 1000 patients. Int J Cardiol. 2011;149:182–185. 20153064 10.1016/j.ijcard.2010.01.005

[9] Hamidi‐Manesh L , Tibby SM , Herman R , Rosenthal E , Qureshi SA , Krasemann T . Influence of balloon size on aortic regurgitation in neonates undergoing balloon aortic valvuloplasty—a retrospective study over an 11‐year period. J Interv Cardiol. 2013;26:200–207. 23406402 10.1111/j.1540-8183.2013.12018.x

[10] Han RK , Gurofsky RC , Lee KJ , Dipchand AI , Williams WG , Smallhorn JF , McCrindle BW . Outcome and growth potential of left heart structures after neonatal intervention for aortic valve stenosis. J Am Coll Cardiol. 2007;50:2406–2414. 18154967 10.1016/j.jacc.2007.07.082

[11] Hochstrasser L , Ruchat P , Sekarski N , Hurni M , von Segesser LK . Long‐term outcome of congenital aortic valve stenosis: predictors of reintervention. Cardiol Young. 2015;25:893–902. 24983130 10.1017/S1047951114001085

[12] Jindal RC , Saxena A , Kothari SS , Juneja R , Shrivastava S . Congenital severe aortic stenosis with congestive heart failure in late childhood and adolescence: effect on left ventricular function after balloon valvuloplasty. Catheter Cardiovasc Interv. 2000;51:168–172. 11025569 10.1002/1522-726x(200010)51:2<168::aid-ccd7>3.0.co;2-c

[13] Kim DW , Raviele AA , Vincent RN . Use of a 3 French system for balloon aortic valvuloplasty in infants. Catheter Cardiovasc Interv. 2005;66:254–257. 16127701 10.1002/ccd.20424

[14] Latiff HA , Sholler GF , Cooper S . Balloon dilatation of aortic stenosis in infants younger than 6 months of age: intermediate outcome. Pediatr Cardiol. 2003;24:17–26. 12370791 10.1007/s00246-002-0187-3

[15] Loomba RS , Bowman JL , Cao Y , Tweddell J , Dearani JA , Simpson PM , Cetta F , Pelech AN . Is aortic valve leaflet morphology predictive of outcome in pediatric aortic valve stenosis? Congenit Heart Dis. 2015;10:552–560. 26219421 10.1111/chd.12278

[16] McElhinney DB , Lock JE , Keane JF , Moran AM , Colan SD . Left heart growth, function, and reintervention after balloon aortic valvuloplasty for neonatal aortic stenosis. Circulation. 2005;111:451–458. 15687133 10.1161/01.CIR.0000153809.88286.2E

[17] Miyamoto T , Sinzobahamvya N , Wetter J , Kallenberg R , Brecher AM , Asfour B , Urban AE . Twenty years experience of surgical aortic valvotomy for critical aortic stenosis in early infancy. Eur J Cardiothorac Surg. 2006;30:35–40. 16725339 10.1016/j.ejcts.2006.03.050

[18] Prijic SM , Vukomanovic VA , Stajevic MS , Bjelakovic BB , Zdravkovic MD , Sehic IN , Kosutic JL . Balloon dilation and surgical valvotomy comparison in non‐critical congenital aortic valve stenosis. Pediatr Cardiol. 2015;36:616–624. 25388630 10.1007/s00246-014-1056-6

[19] Robinson BV , Brzezinska‐Rajszys G , Weber HS , Ksiazyk J , Fricker FJ , Fischer DR , Ettedgui JA . Balloon aortic valvotomy through a carotid cutdown in infants with severe aortic stenosis: results of the multi‐centric registry. Cardiol Young. 2000;10:225–232. 10824903 10.1017/s104795110000915x

[20] Rossi RI , Manica JL , Petraco R , Scott M , Piazza L , Machado PM . Balloon aortic valvuloplasty for congenital aortic stenosis using the femoral and the carotid artery approach: a 16‐year experience from a single center. Catheter Cardiovasc Interv. 2011;78:84–90. 21234922 10.1002/ccd.22938

[21] Hayden JA , van der Windt DA , Cartwright JL , Cote P , Bombardier C . Assessing bias in studies of prognostic factors. Ann Intern Med. 2013;158:280–286. 23420236 10.7326/0003-4819-158-4-201302190-00009

[22] Guyot P , Ades AE , Ouwens MJ , Welton NJ . Enhanced secondary analysis of survival data: reconstructing the data from published Kaplan‐Meier survival curves. BMC Med Res Methodol. 2012;12:9. 22297116 10.1186/1471-2288-12-9 PMC3313891

[23] Karamlou T , Shen I , Alsoufia B , Burch G , Reller M , Silberbach M , Ungerleider RM . The influence of valve physiology on outcome following aortic valvotomy for congenital bicuspid valve in children: 30‐year results from a single institution. Eur J Cardiothorac Surg. 2005;27:81–85. 15621475 10.1016/j.ejcts.2004.10.044

[24] Reich O , Tax P , Marek J , Razek V , Gilik J , Tomek V , Chaloupecky V , Bartakova H , Skovranek J . Long term results of percutaneous balloon valvoplasty of congenital aortic stenosis: independent predictors of outcome. Heart. 2004;90:70–76. 14676248 10.1136/heart.90.1.70 PMC1768014

