# Supplementary material for: Surgical Valvotomy Versus Balloon Valvuloplasty for Congenital Aortic Valve Stenosis: A Systematic Review and Meta‐Analysis
Source: J Am Heart Assoc. 2016 Aug 8;5(8):e003931. doi: 10.1161/JAHA.116.003931 (PMC5015309; doi:10.1161/JAHA.116.003931)
Supplement: Supplementary file 1 — Table S1. Full Hayden Risk of Bias Scoring Figure S1. Outcomes by risk of bias. Kaplan‐Meier curve for survival (A and B), freedom from aortic valve replacement (C and D), and freedom from reintervention by Hayden risk of bias score (E and F). Scores were dichotomized to low risk (Hayden bias score of 6) or high risk (Hayden bias score >6). [file JAH3-5-e003931-s001.pdf]

SUPPLEMENTAL MATERIAL

Table S1: Full Hayden risk of bias scoring

Article	Participation	Attrition	Prognostic Factor Measurement	Outcome measurement	Confounding	Analysis and reporting
**Alexiou**	1	1	1	1	1	2
**Bhabra**	1	1	1	1	1	1
**Brown**	1	1	1	1	2	1
**Crespo**	1	2	1	1	1	1
**Elshershari**	1	1	2	2	2	1
**Ewert**	1	1	1	1	1	1
**Hamidi-Manesh**	1	1	1	1	1	2
**Han**	1	1	1	1	1	1
**Hochstrasser**	1	2	1	1	1	1
**Jindal**	1	1	1	1	1	1
**Kim**	1	1	1	1	1	1
**Latiff**	1	1	1	1	1	1
**Loomba**	1	1	1	1	1	1
**McCrindle**	1	1	1	1	1	1
**McElhinney**	1	1	1	1	2	1
**Miyamoto**	1	1	1	1	1	1
**Prijic**	1	1	1	1	1	1
**Robinson**	1	1	1	1	1	1
**Rossi**	1	1	1	1	1	1
**Siddiqui**	1	1	1	1	1	1

Hayden bias scores by domain with 1 representing low risk of bias, 2 representing moderate risk of bias and 3 representing a high risk of bias.

Figure S1: Outcomes by risk of bias. Kaplan Meier curve for survival (**A,B**), freedom from aortic valve replacement (**C,D**) and freedom from re-intervention by Hayden risk of bias score (**E,F**). Scores were dichotomized to low risk (Hayden bias score of 6) or high risk (Hayden bias score >6).

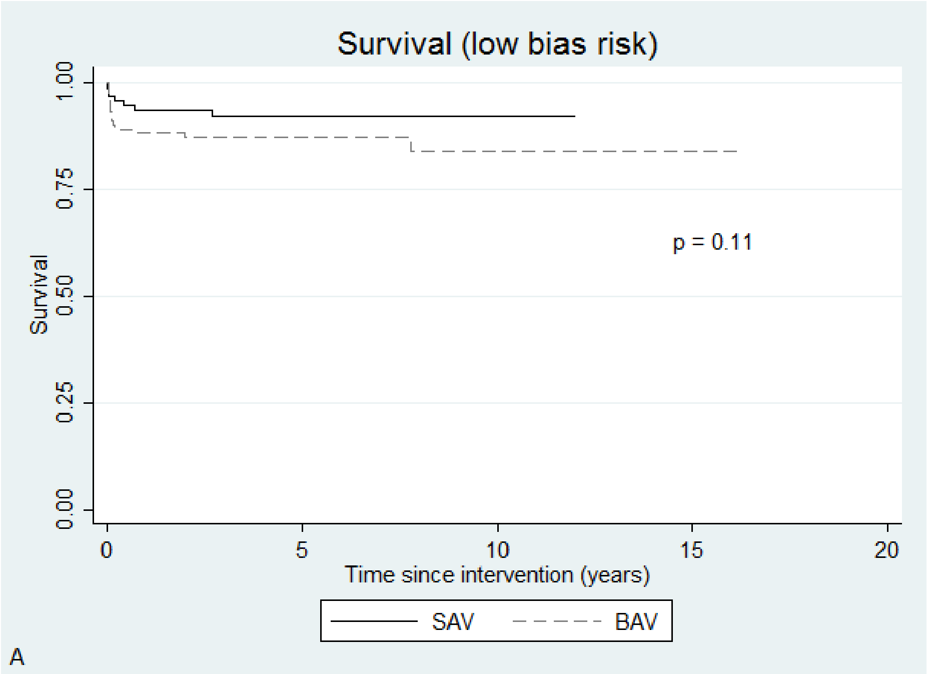

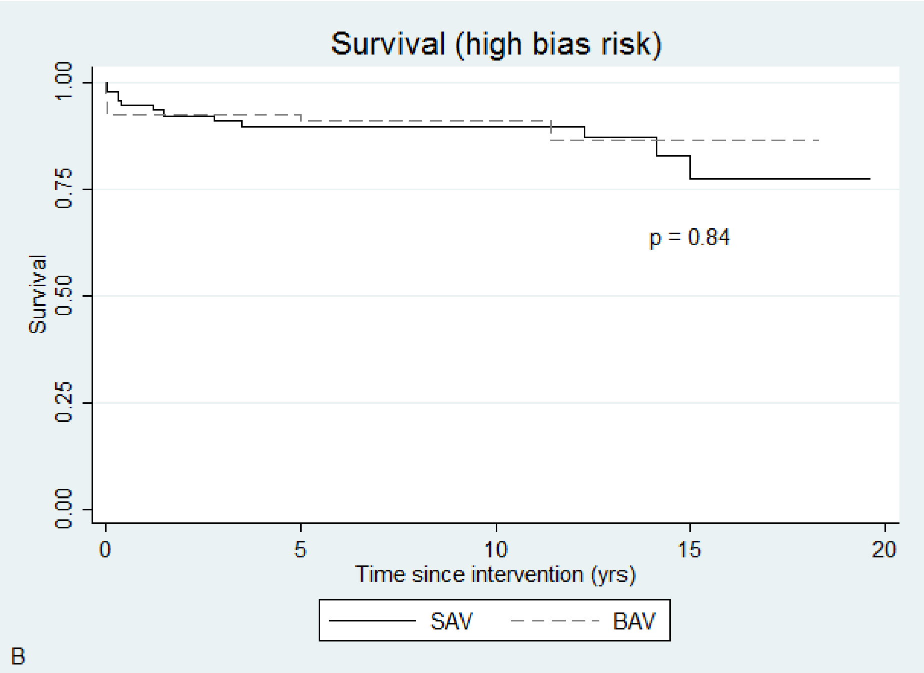
Survival (high bias risk)
Survival
1.00
0.75
0.50
0.25
0.00
p = 0.84
0
5
10
15
20
Time since intervention (yrs)
SAV
BAV
B

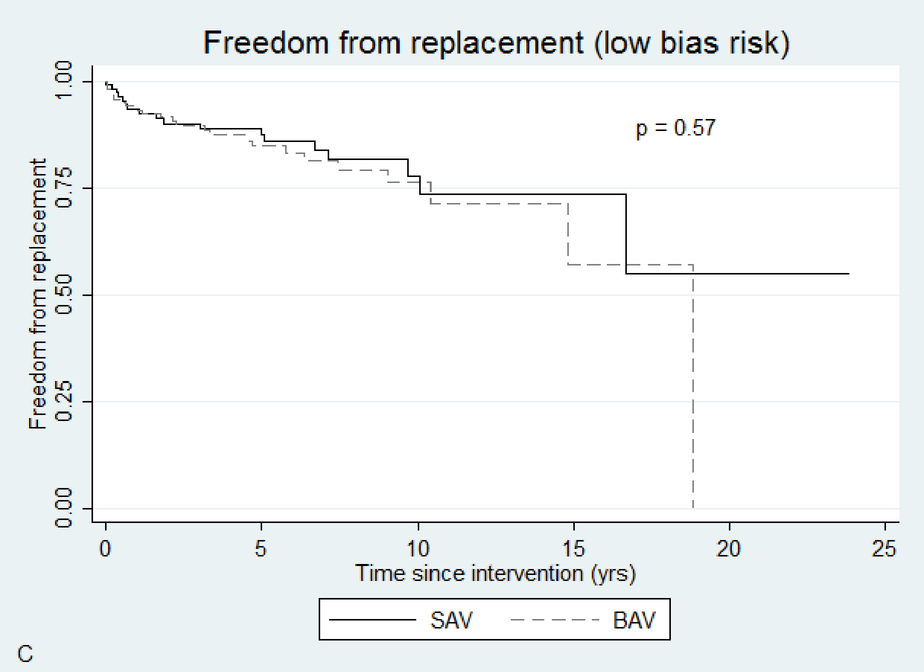
Freedom from replacement (low bias risk)
Freedom from replacement
1.00
0.75
0.50
0.25
0.00
p = 0.57
0
5
10
15
20
25
Time since intervention (yrs)
SAV
BAV
C

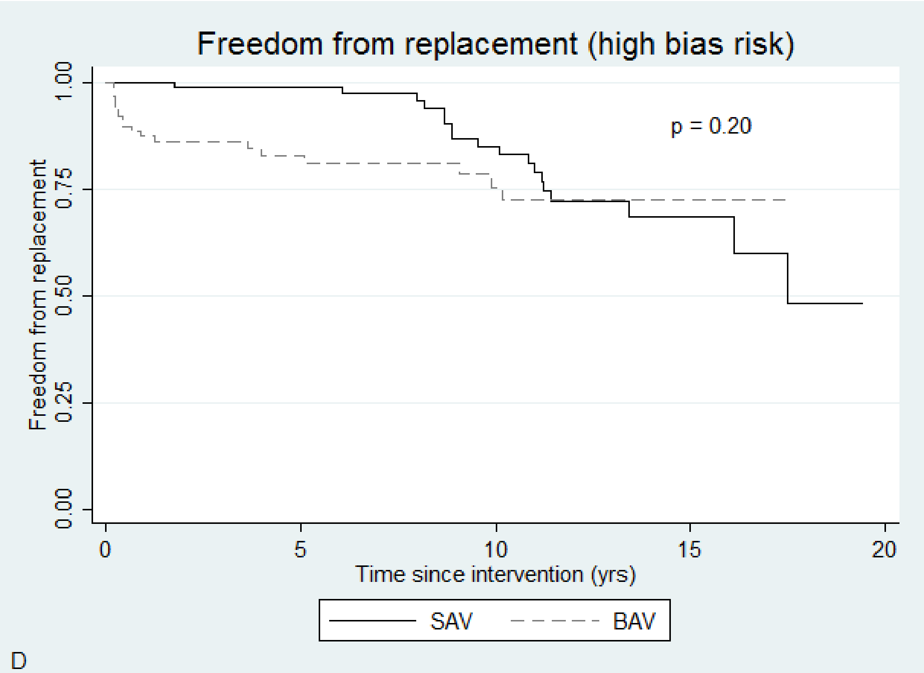
Freedom from replacement (high bias risk)
Freedom from replacement
1.00
0.75
0.50
0.25
0.00
p = 0.20
0
5
10
15
20
Time since intervention (yrs)
SAV
BAV
D

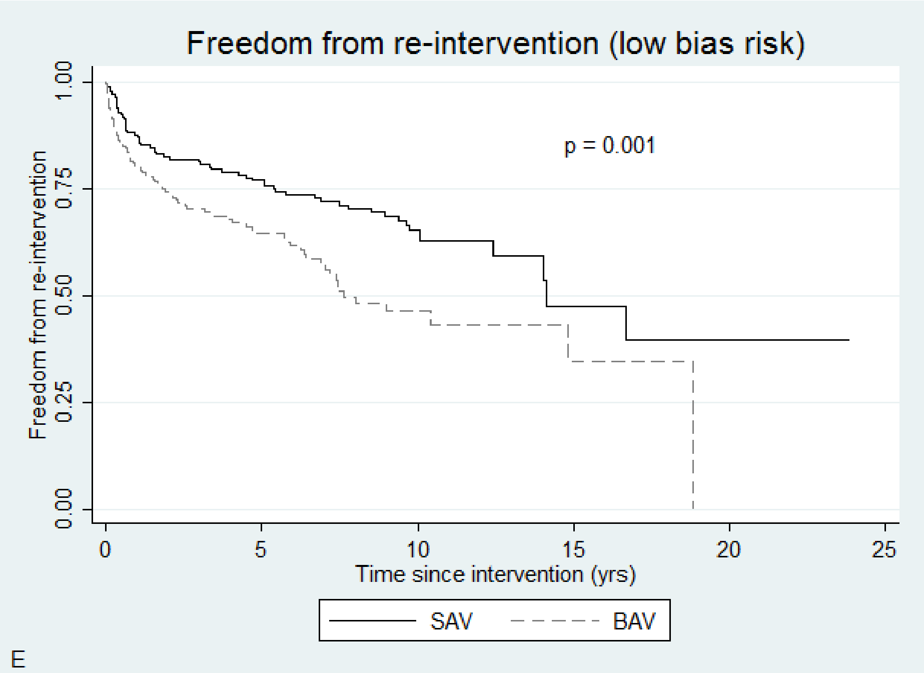
Freedom from re-intervention (low bias risk)
Freedom from re-intervention
1.00
0.75
0.50
0.25
0.00
p = 0.001
0
5
10
15
20
25
Time since intervention (yrs)
SAV
BAV
E

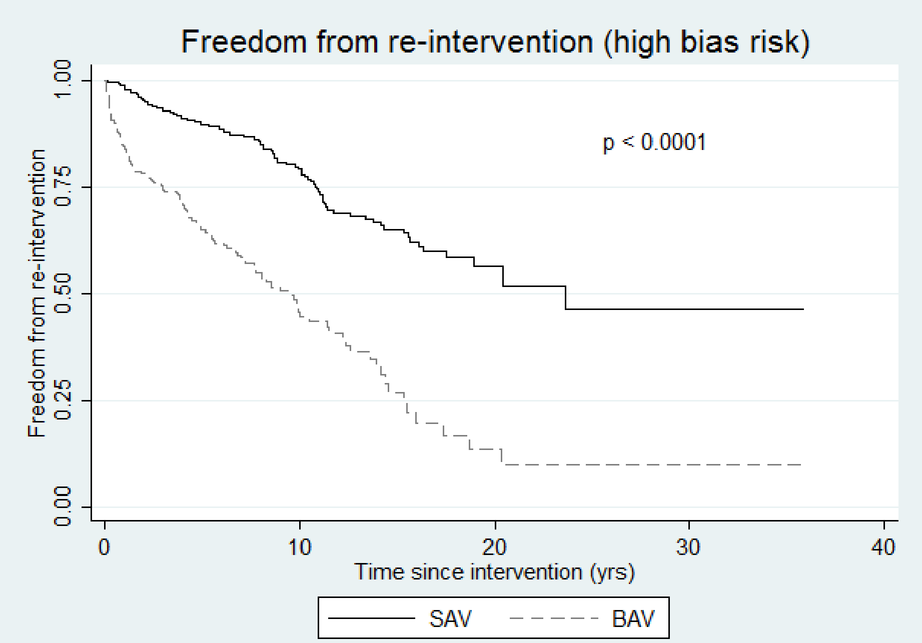

Freedom from re-intervention (high bias risk)
Freedom from re-intervention
1.00
0.75
0.50
0.25
0.00
p < 0.0001
0
10
20
30
40
Time since intervention (yrs)
SAV
BAV

F
